# Transfer learning for EEG-based BCIs: a comparative evaluation and optimization of data alignment methods

**DOI:** 10.3389/fnsys.2026.1840121

**Published:** 2026-06-11

**Authors:** Soha Galalaldin Ahmed, Medha Mohan Ambali Parambil, Rafat Damseh, Salah Bouktif, Fady Alnajjar, Abdelkader Nasreddine Belkacem

**Affiliations:** 1 Department of Computer Science and Software Engineering, College of Information Technology, United Arab Emirates University, Al Ain, United Arab Emirates; 2 Department of Computer and Network Engineering, College of Information Technology, United Arab Emirates University, Al Ain, United Arab Emirates

**Keywords:** brain-computer interface, coral, cross-subject generalization, data alignment, EEG, Euclidean Alignment, Riemannian Procrustes Analysis, transfer learning

## Abstract

**Background:**

This paper addresses a critical challenge in developing practical EEG-based brain-computer interfaces (BCIs): enhancing cross-subject generalization by mitigating individual differences in brain signals. How can we effectively leverage data from existing subjects to improve performance for a new user with minimal subject-specific calibration?

**Methods:**

We systematically compare and optimize three prominent data alignment techniques, Riemannian Procrustes Analysis (RPA), Euclidean Alignment (EA), and Correlation Alignment (CORAL), designed to transform EEG data from multiple source subjects and a target subject into a common representation space, mitigating variability.

**Evaluation:**

We employed leave-one-subject-out cross-validation (LOSO-CV) framework on EEG-based attention decoding data to empirically evaluate the effectiveness of each alignment method compared to a baseline condition with no alignment. Key parameters, specifically the regularization parameter α for EA, were optimized to maximize cross-subject transfer performance.

**Results:**

The study demonstrates that alignment methods improve classification accuracy compared to the baseline. Notably, EA evaluated at α = 100 the scaling value at which the largest fraction of subjects attained their best accuracy in our parameter sweep yielded the largest mean improvement, increasing classification accuracy by 3.44% over the no alignment baseline (paired *t*(17)≈2.48, *p*≈0.024; Cohen's *d*_*z*_≈0.59; 95% confidence interval for the mean improvement [0.52%, 6.36%]). Because this α value was identified from the same sweep that produced the per-subject accuracies, this estimate together with the per-subject “best-parameter” results should be interpreted as an oracle sensitivity-analysis upper bound on subject-specific tuning rather than as a leakage-free LOSO estimate. While optimized EA showed the best mean performance, the analysis also demonstrated subject-specific differences in the most ideal alignment strategy.

**Conclusion:**

This comparison framework quantifies the benefits of different alignment approaches and highlights the valuable contribution of parameter optimization, particularly for EA.

**Significance:**

These results indicate the potential of optimized alignment techniques, EA in particular, to significantly enhance cross-subject transfer learning in EEG-based BCIs. This has practical ramifications for methodology selection and tuning, and maps a path toward more robust and generalizable BCI systems requiring less subject-specific calibration for real-world applications.

## 1 Introduction

A brain-computer interface (BCI) provides an immediate interface between the human brain and an external machine, mapping brain signals reflecting user intentions or cognitive states (Lotte et al., 2015). Electroencephalography (EEG) remains the most popular input modality for non-invasive BCIs due to the fact that it is portable, harmless, and provides high temporal resolution. These systems have great promise for applications in neurorehabilitation, assistive technologies for individuals with disabilities, and cognitive monitoring tasks such as attention tracking and emotion detection (Belkacem et al., 2020; Jamil et al., 2021). Standard BCI paradigms utilize unique neural signatures, e.g., sensorimotor rhythms modulated by motor imagery (MI) (Kang et al., 2009; Tu and Sun, 2012) or event-related potentials (ERPs) evoked by particular stimuli (Reichert et al., 2020). This article considers attention decoding with EEG signals.

One of the biggest challenges to making BCIs practical in daily life is high inter-subject variability in brain signals across individuals. This variability makes it difficult to develop models that can perform well for any individual without requiring time-consuming and lengthy calibration sessions for each individual (Jeng et al., 2020). Inter-subject variability occurs since individuals' brains vary in anatomy, neural adaptability, and personal styles of cognition (Lebreton et al., 2019). Even if two people perform the same task, the EEG traces might look completely different. Traditional approaches try to tackle this by creating personalized models for each user. However, this method has limitations, it takes hours of calibration and isn't feasible in situations where BCIs need to work right out of the box (Lebreton et al., 2019; Belkacem et al., 2023).

**Figure 1:**
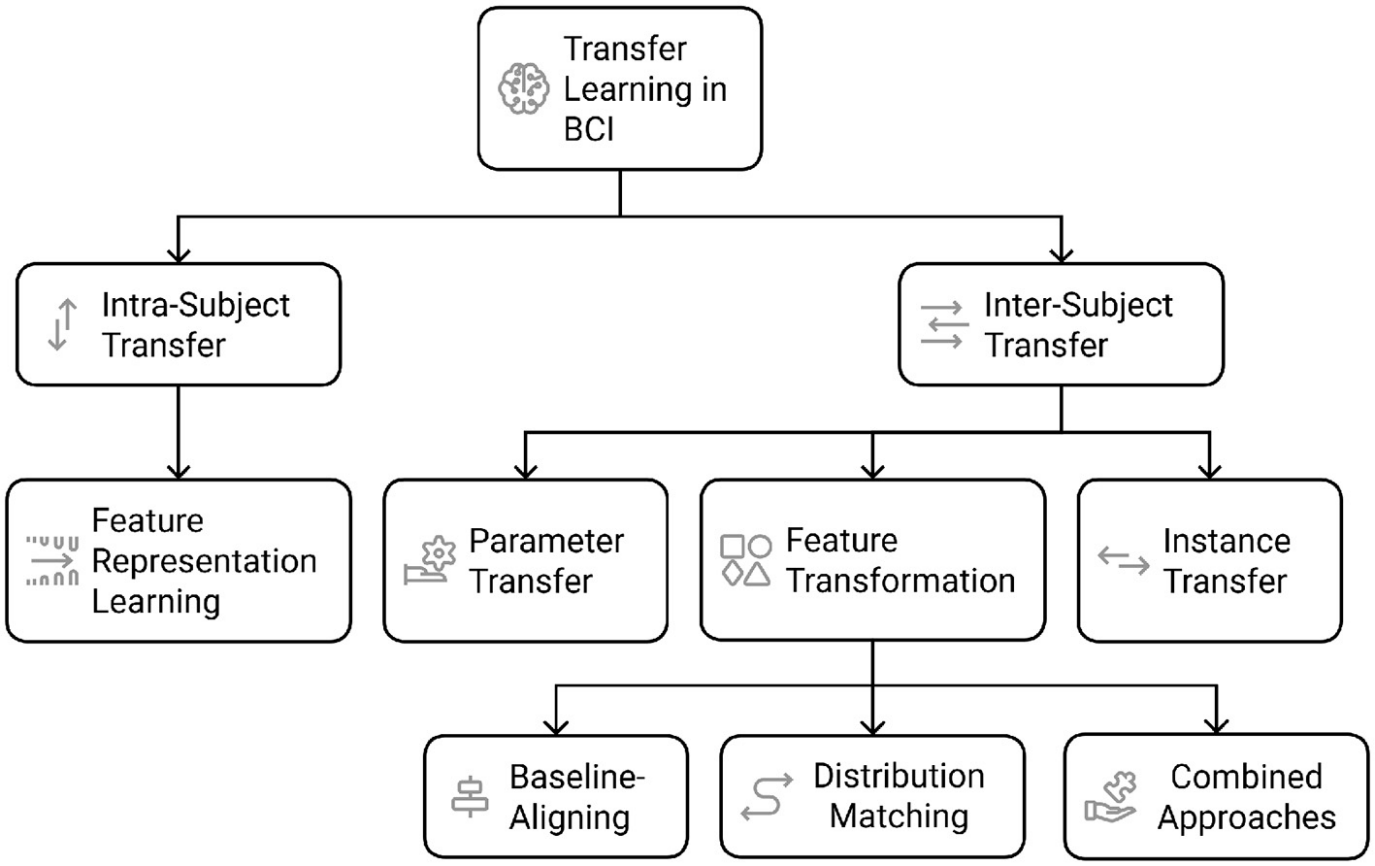
Taxonomy of transfer learning in BCI: This framework categorizes intra-subject and inter-subject transfer approaches.

To overcome these limitations and reduce the calibration burden, transfer learning (TL) has emerged as a powerful paradigm. TL aims at leveraging knowledge gained from existing 'source' subjects to improve learning performance on a new 'target' subject, thereby minimizing the need for extensive target-specific data (Lotte et al., 2018). TL encompasses various strategies applicable to BCIs, broadly categorized into approaches that adapt models across sessions for the same subject (intra-subject) or adapt models across different subjects (inter-subject), as illustrated in the taxonomy shown in Figure 1.

A key strategy within inter-subject TL for EEG-BCIs is data alignment. The core idea is to mathematically transform the EEG data from different subjects into a shared feature space, thereby reducing the distributional discrepancies caused by inter-subject variability and making the data more comparable for model training (Sun et al., 2016). Several alignment techniques have been proposed, often operating in different mathematical domains. For instance, methods like Riemannian Alignment (RA) operate on covariance matrices within the Riemannian manifold (Zanini et al., 2017). Alternatively, He and Wu (2019) proposed an effective Euclidean Alignment (EA) approach that directly aligns and transforms raw EEG trials in the Euclidean space, with promising use cases for transfer learning in MI and ERP tasks. Other approaches, like Correlation Alignment (CORAL) (Sun et al., 2016), focus specifically on aligning the second-order statistics (covariances) of the source and target data features.

While individual alignment methods like the EA approach proposed by He and Wu (2019) have demonstrated potential, there is a need for a systematic comparison of different alignment philosophies, such as those based on Riemannian geometry (like Riemannian Procrustes Analysis, RPA), direct Euclidean transformation, and statistical feature alignment (CORAL), to understand their relative strengths, weaknesses, and optimal application within the context of EEG-BCIs, particularly for tasks like attention decoding (Reichert et al., 2020; Chugh and Aggarwal, 2024). Furthermore, methods like EA and CORAL often involve hyperparameters (e.g., regularization terms) whose optimal settings for maximizing cross-subject transfer performance warrant specific investigation.

This study tests three hypotheses. First, data alignment improves leave-one-subject-out (LOSO) transfer performance over no alignment. Second, parameter choice materially affects alignment performance, especially for EA and CORAL. Third, the best alignment strategy is subject-dependent rather than universal. Accordingly, the contribution of this work is not a new alignment algorithm, but a controlled comparative evaluation and parameter sensitivity study of lightweight alignment methods for covert spatial attention decoding.

This paper undertakes such a comparative study and optimization of three distinct data alignment methods: RPA, EA, and CORAL. Contributions from this study are:

- We implement and apply these three alignment methods within a unified transfer learning framework for EEG-BCI decoding.
- We systematically evaluate their effectiveness in improving cross-subject generalization using a leave-one-subject-out cross-validation (LOSO-CV) scheme.
- We optimize key parameters for EA and CORAL to maximize their transfer performance.
- We analyze subject-specific results to provide insights into which methods might be preferable under different circumstances.

By comparing these diverse approaches, we aim to provide a clearer understanding of their impact on transfer learning for EEG-BCIs and offer practical guidance for method selection and tuning in real-world scenarios.

The remainder of this paper is organized as follows: Section II introduces the alignment methods (RPA, EA, CORAL). Section III describes the dataset and experimental setup. Section IV presents the comparative results and parameter optimization findings. Finally, Section V discusses the implications and concludes the paper.

## 2 Related works

This section reviews previous work on transfer learning techniques used in EEG-based brain-computer interfaces (BCIs).

While some studies focused on transferring knowledge across sessions for the same user, this work concentrates on transfer learning between different subjects. Table 1 summarizes prior transfer learning approaches in EEG-based BCIs and situates the present comparative evaluation within the literature.

**Table 1:** Transfer learning approaches for EEG-based studies.

Paper	Target domain	Calibration data	Transfer learning approach
(Kang et al. 2009)	Existing subject	N/A	Feature-representation
(Tu and Sun 2012)	Existing subject	Whole data	Feature-representation
(Özdenizci et al. 2020)	Existing subject	Whole data	Feature-representation
(Alamgir et al. 2010)	Existing subject	Whole data	Parameter
(Jayaram et al. 2016)	Existing subject	Whole data	Parameter
(Zhang et al. 2015)	Existing subject	Whole data	Distribution-matching
(Reuderink et al. 2011)	New subject	Task-relevant data	Baseline-aligning
(Zanini et al. 2017)	New subject	Task-relevant data	Baseline-aligning
(He and Wu 2019)	New subject	Task-relevant data	Baseline-aligning
(Rodrigues et al. 2018)	New subject	Task-relevant data	Distribution-matching
(Dai et al. 2019)	New subject	Task-relevant data	Distribution-matching
(Zhang and Wu 2020)	New subject	Task-relevant data	Baseline-aligning & Distribution-matching
(Li et al. 2019b)	New subject	Task-relevant data	Distribution-matching
(Dagois et al. 2018)	New subject	Task-relevant data	Instance
(Zhang et al. 2019b)	New subject	Task-relevant data	Instance
(Zhang et al. 2019a)	New subject	Task-relevant data	Feature-representation
(Jeon et al. 2021)	New subject	Task-relevant data	Feature-representation
(Li et al. 2019a)	New subject	Task-relevant data	Distribution-matching
(Bolagh and Clifford 2017)	New subject	Pre-trial/resting data	Instance
(Wei et al. 2018)	New subject	Pre-trial/resting data	Parameter

Early research in this area focused on learning features that remain stable across subjects. This is known as feature representation learning. For example, Kang et al.(Kang et al., 2009) proposed a weighted common spatial filter (CSF) that worked across subjects. Tu and Sun (2012) later extended this by creating both shared CSFs for all users and personalized filters for each individual. Özdenizci et al. (2020) applied convolutional neural networks and adversarial training to learn subject-invariant features. Similarly, Zhang et al. (2019a) and Jeon et al. (2021) used deep learning to extract consistent patterns across users.

As research evolved, a new direction called parameter-transfer learning was introduced. This approach trains models on data from multiple users and then adjusts some parameters to handle individual differences. For instance, Alamgir et al. (2010) proposed a method that learns shared model parameters while fine-tuning for each subject. Jayaram et al. (2016) showed that pre-trained models can be adapted to new users using calibration data.

More recently, feature-transformation methods have gained attention. These methods reduce subject-specific differences by transforming the features. There are two main types: baseline-aligning and distribution-matching. Baseline-aligning removes individual-specific baselines to make the data more uniform (Reuderink et al., 2011; Zanini et al., 2017; He and Wu, 2019). Distribution-matching transforms features so that the distributions across users become more similar (Li et al., 2019b; Zhang et al., 2015; Rodrigues et al., 2018; Dai et al., 2019; Li et al., 2019a). Some studies combine both strategies for better results (Zhang and Wu, 2020).

Another strategy is instance-transfer, where more weight is given to labeled data from source subjects that are more similar to the target subject. This weighting is often based on the distance between their data distributions (Dagois et al., 2018; Zhang et al., 2019b).

Although these methods have shown good performance in controlled lab settings, most assume that labeled data is available for all classes. This assumption doesn't hold in real-world scenarios, like drowsiness or seizure detection, where collecting enough labeled data for every new user may not be possible.

Recent EEG transfer learning research has increasingly shifted toward deep representation learning, including domain-adversarial networks, self-supervised and contrastive learning, transformer-based models, and foundation model paradigms. While these approaches can capture non-linear subject variability and benefit from large-scale pretraining, they typically require substantially larger datasets, greater computational resources, and more complex training pipelines. In contrast, alignment-based methods remain attractive in small-sample EEG settings due to their simplicity, interpretability, and low calibration cost.

Recent work has also extended alignment methods beyond inter-subject transfer. Transfer EA (Vishwanath et al., 2025) enables inter-dataset and cross-species transfer, demonstrating improved classification between human and mouse EEG. Covariance-based harmonization frameworks (Mellot et al., 2023) decompose alignment into re-centering, re-scaling, and rotation, highlighting re-centering as critical for cross-population generalization. A recent review (Wu, 2025) further consolidates these developments and expands the scope of alignment to MEG, intracranial EEG, and cross-modality applications.

Together, these advances show that alignment remains an active and evolving area, increasingly used as a component within broader transfer pipelines. Accordingly, the present study focuses on a controlled comparison of classical alignment operators in the inter-subject setting, while providing insights relevant to emerging inter-dataset and cross-species applications.

We also went through the studies that used or cited the dataset by Reichert et al. (2020), as shown in Table 2. Some of these studies applied classifiers such as decision trees, SVMs, LSTMs, and CNNs to decode cognitive states from EEG, reporting performance that ranged from moderate to high accuracy. Others used the dataset primarily to support claims related to stimulus design, eye movement artifacts (EOG), or covert attention mechanisms. A few papers focused more on theoretical exploration or methodology validation rather than model performance. These prior works highlight the dataset's utility in both applied machine learning and cognitive neuroscience contexts.

**Table 2:** Studies that used or cited the dataset.

Year	Author(s) and reference	Results/notes
2021	(Gutierrez-Martinez et al., 2021)	Review paper
2021	(Shahbakhti et al., 2021)	TPR: 98.52% for EOG
2022	(Fernández-Rodŕıguez et al., 2022)	Cited for stimulus impact discussion
2022	(Wang et al., 2022)	Cited for stimulus features study
2023	(Abdel-Samei et al., 2023)	Cited for EOG
2022	(Xu et al., 2022)	
2024	(Reichert et al., 2024)	
2024	(Ron-Angevin et al., 2024)	Cited for covert attention
2024	(Zhou et al., 2024)	
2021	(Altan et al., 2021)	Decision Tree: 89.24%, k-NN: 70.10%, MLP: 64.86%, SVM: 62.92%
2024	(Chugh and Aggarwal, 2024)	LSTM: 92.79%, CCA: 88.8%, SVM: 61.76%
2024	(Yao et al., 2024)	
2024	(Paul et al., 2024)	
2023	(Van de Wauw et al., 2023)	
2022	(An et al., 2022)	MSSA-TN: 33.33%, CCN: 22.92%, SVM: 25.00%, KNN: 27.08%, RF: 37.50%, LDA: 12.50%
2022	(Reichert et al., 2022)	Cited. Same authors' paper
2023	(Kæseler, 2023)	
2022	(Aggarwal et al., 2022)	SVM: 66.6%, Gradient boosting: 67.9%, CNN: 70.0%
2021	(Honźık, 2021)	CNN with two conv layers: 52.31%

We selected this dataset for our study because it targets covert spatial attention shifts, a task relevant to many real-world BCI applications. Moreover, it contains rich time-resolved EEG data recorded across multiple subjects, making it ideal for evaluating and comparing alignment-based transfer learning strategies. The dataset also provides enough subject diversity and trials to meaningfully assess cross-subject generalization performance, which is the central aim of our work.

## 3 Methodology

**Figure 2:**
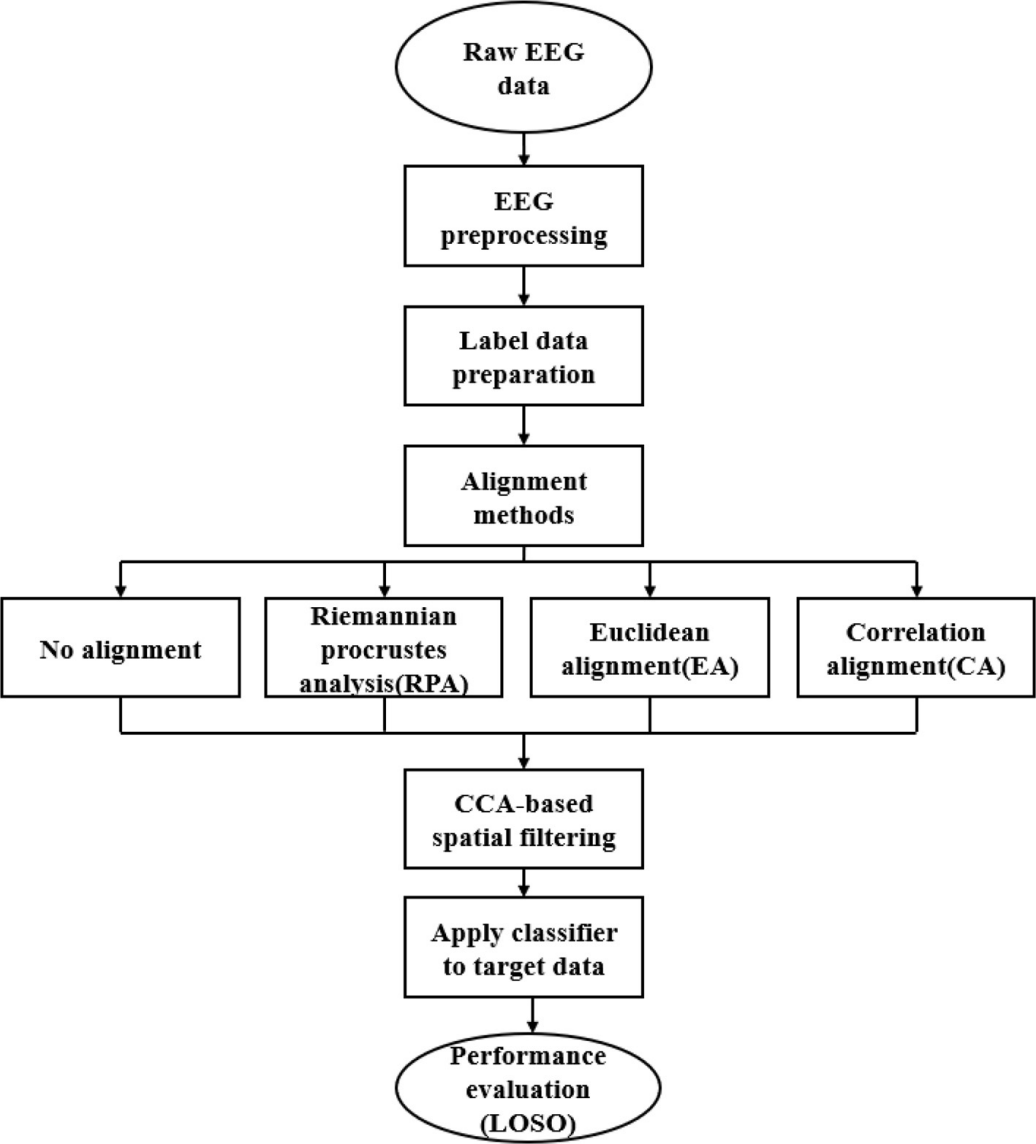
Methodological pipeline of the study: the stepwise process of the study, including EEG pre-processing, label preparation, alignment methods, spatial filtering, classification, and performance evaluation using LOSO validation.

The current study focuses on evaluating feature-transformation techniques and their optimization strategies for addressing inter-subject variability in EEG-based BCIs. The methodology comprises several key components, including dataset preparation, pre-processing, alignment methods, parameter optimization, and evaluation metrics. The methodological pipeline overview of the work is shown in Figure 2.

### 3.1 Dataset

This research used the data set from a study by Reichert et al. (2020) with a group of participants in Germany. The study involved 18 healthy individuals who participated in a series of visual attention experiments. The researchers recorded brain signals while participants looked at visual cues, specifically focusing on how people shift their attention without moving their eyes. This type of attention shift, where someone focuses on different areas in their field of vision while keeping their eyes still, helps us understand how the brain processes visual information.

**Figure 3:**
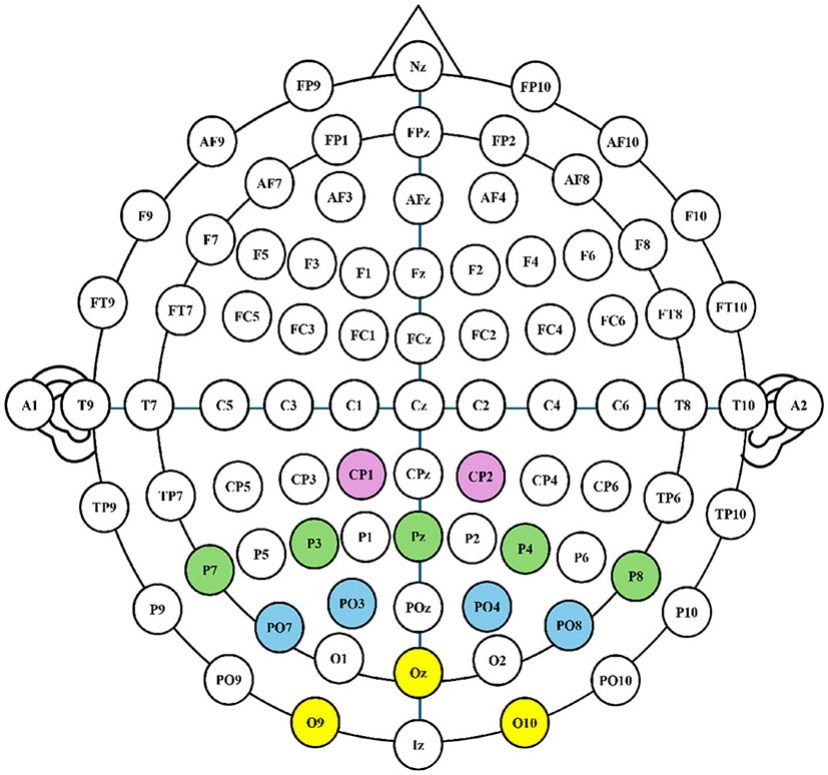
Electrode placements based on the 10–20 system, highlighting the 14 parieto-occipital channels selected for attention decoding in this study.

The brain activity recording focused on specific areas of interest. Electrode placements are shown in Figure 3.

Although the original set-up used 30 electrodes, the study focused on 14 key channels in the back of the head, an area that is particularly important for processing visual information. These channels included O9, O10, CP1, CP2, Pz, P3, P4, P7, P8, PO3, PO4, PO7, PO8, and Oz as shown in Figure 3. The researchers chose these specific locations because they're known to be most active when people are processing visual information and shifting their attention. During the experiment, participants went through seven different experimental runs. They started with simple tasks where they had to focus their attention on either a green cross, which represented “yes,” or a red cross, which meant “no.” As they progressed through the experiment, the tasks became more complex. In later runs, participants had to answer actual questions by focusing on either the green or red cross, and in the final run, they even shared their personal opinions using the same method.

**Figure 4:**
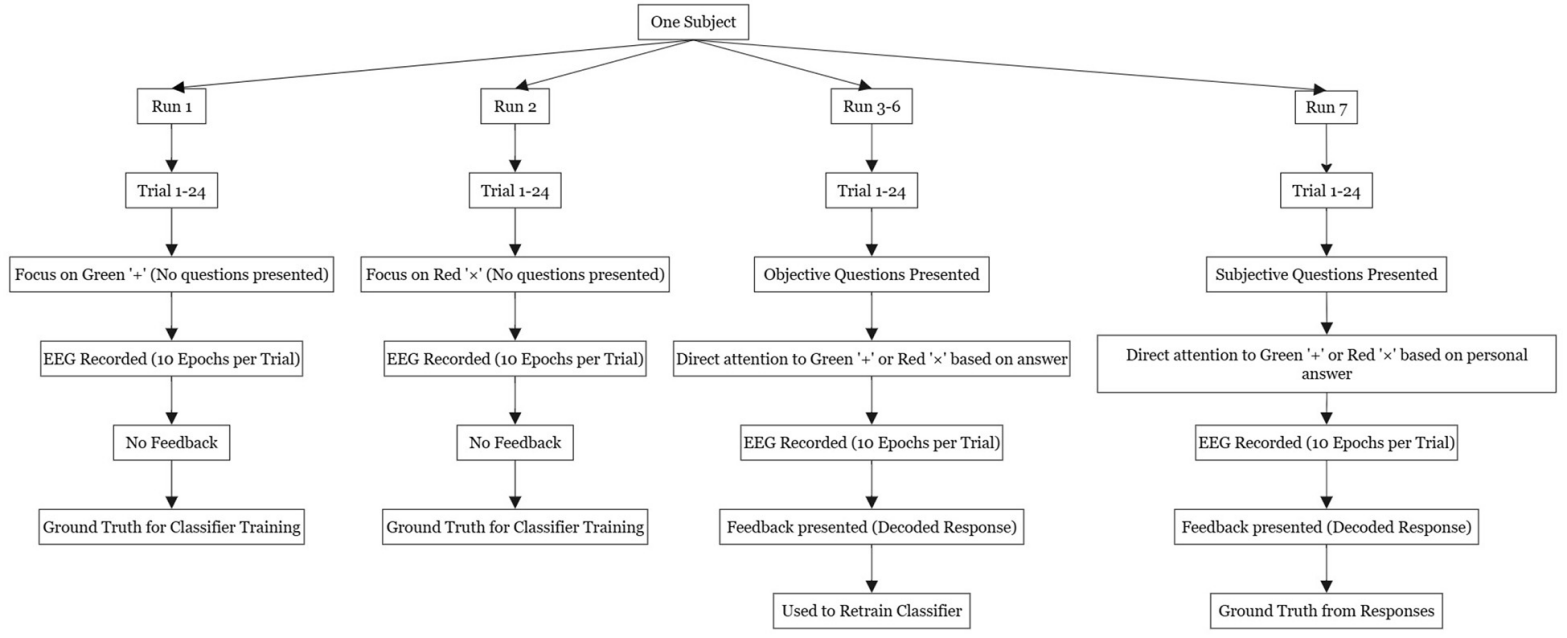
Experiment procedure.

Each person completed 24 trials in every run, which means they did 168 trials in total. Within each trial, the researchers collected data from 10 different time points, giving them 1,680 separate pieces of data from each participant. Figure 4 provides a thorough description of the experimental steps used by the authors to create the dataset.

The researchers presented the visual cues, a red “x” cross and a green “+” cross, on opposite sides of a screen. While recording brain signals at 250 Hz, they saved all this information in MATLAB files. For every trial, they kept track of many different aspects: exactly when and where they showed the crosses, which cross the participant was supposed to pay attention to, how the participant responded, and even details like how far the crosses were from the center of the screen and how big they were.

We selected this dataset because it is well-annotated and trial-dense for a controlled covert spatial attention benchmark, although its cohort size is modest at 18 subjects. This makes it suitable for LOSO evaluation of alignment methods, but conclusions should be interpreted as specific to this paradigm until validated on larger and more diverse datasets.

### 3.2 EEG pre-processing

The raw EEG data was also undergone a sequence of pre-processing steps to enhance signal quality and prepare it for decoding. The EEG signals were initially re-referenced to the left and right mastoid average to eliminate hemispheric biases caused by asymmetric placements of the electrodes. Second, channel selection was done through retaining the 14 parieto-occipital channels, which are of most crucial importance to visual attention tasks.

Following channel selection, the signals were filtered using a 4th-order zero-phase IIR Butterworth band-pass filter with a frequency range of

1.0–12.5 Hz. This filtered out high-frequency noise and retained the EEG frequency bands of interest for cognitive and attentional processing. The EEG data was then resampled to 50 Hz for reducing the computational complexity and increasing efficiency in processing. During the epoching step, EEG data were separated into epochs of 750 ms, which is equivalent to 38 time points at a sampling rate of 50 Hz. Epoching was initiated at the onset of the stimulus so that EEG data would be synchronized with experimental triggers. Then, 10 epochs for 10 individual stimuli were concatenated to result in a single labeled trial dataset. Trials were divided by the target direction (e.g., left *vs*. right visual cue) to produce the final dataset used for model building and decoding.

The pre-processing pipeline follows the original Reichert et al. analysis on the same dataset (Reichert et al., 2020), including 1.0–12.5 Hz bandpass filtering and resampling to 50 Hz, to ensure that any performance differences are attributable to alignment rather than pre-processing. This pass-band is appropriate for the neural targets of the task: lateralized covert spatial attention is primarily reflected in low-frequency activity, notably the N2pc component (200–300 ms) and posterior alpha-band dynamics (8–12 Hz), both largely contained below ~12 Hz. The upper cutoff was chosen to capture the full alpha range while minimizing leakage into higher frequencies.

Restricting the analysis to this band also reduces contamination from electromyographic activity and microsaccades, which disproportionately affect higher frequencies and are particularly relevant in a gaze-independent paradigm. We therefore treat this pre-processing as a task-specific design choice aligned with the benchmark, rather than a universally optimal setting. While higher-frequency activity may be informative in other attention paradigms, evaluating broader spectral ranges remains an important direction for future work.

### 3.3 Alignment methods

To address inter-subject variability, we experimented with the following setups: No Alignment (baseline) and three alignment methods: RPA, EA, and CORAL. These methods align EEG signals between subjects by transforming features into a shared representation space. The *No alignment* strategy was the control, in which the EEG data was used without converting them. This provided a benchmark to measure the efficacy of the alignment strategies. *RPA* equates the covariance matrices of EEG data between subjects to a common manifold. The method involves computing the covariance matrices of both source and target data, followed by whitening and alignment transformations. The whitening matrix ensures that the source data has an identity covariance, and the alignment matrix maps the whitened source data to the target space. *EA* transforms EEG data by centering it around a common mean. First, the mean of the EEG data for each subject is computed. Then, the data is aligned by subtracting the subject-specific mean and adding the grand mean of all subjects. This transformation ensures that the data from different subjects is centered around a uniform meanwhile retaining the variance structure. *CORAL* matches the covariance structures of the source and target EEG data. The target covariance matrix is regularized, and a transformation is derived to align the covariance of the source data to that of the target data. CORAL minimizes the differences in second-order statistics (covariance) between domains. Specifically, the source features are whitened using the inverse square root of the source covariance matrix and then recolored using the square root of the target covariance matrix, thereby aligning the covariance structure of the source domain to that of the target domain.

### 3.4 Optimization algorithms

To optimize the performance of each baseline alignment method, we introduced a regularization variable for each method.

#### 3.4.1 regularized RPA (rRPA)

To improve the performance of the RPA alignment method, we introduced regularized RPA (rRPA) with a tunable parameter α that balances the contribution of the sample covariance matrix **S** and a scaled identity matrix **I**. The regularized covariance matrix is defined as:


$${\text{C}}_{\text{r}\text{e}\text{g}\text{u}\text{l}\text{a}\text{r}\text{i}\text{z}\text{e}\text{d}}=\left(1-\alpha \right)\cdot \text{S}+\alpha \cdot \frac{\text{t}\text{r}\text{a}\text{c}\text{e}\left(\text{S}\right)}{n_{\text{c}\text{h}\text{a}\text{n}\text{n}\text{e}\text{l}\text{s}}}\cdot \text{I},$$

where α∈[0, 1] controls the shrinkage intensity. For α = 1, the formula simplifies to:


$${\text{C}}_{\text{r}\text{e}\text{g}\text{u}\text{l}\text{a}\text{r}\text{i}\text{z}\text{e}\text{d}}=\frac{\text{t}\text{r}\text{a}\text{c}\text{e}\left(\text{S}\right)}{n_{\text{c}\text{h}\text{a}\text{n}\text{n}\text{e}\text{l}\text{s}}}\cdot \text{I}.$$

This setting completely regularizes the covariance matrix by disregarding the sample covariance **S**, resulting in an isotropic covariance matrix. Such a matrix assumes that all channels are uncorrelated with equal variance across channels.

#### 3.4.2 Role of λ and α par and EA Alignment Methods

##### 3.4.2.1 λ in CORAL (regularization parameter)

The λ parameter in CORAL controls the regularization of the target domain covariance matrix. Regularization is crucial to balance the use of the actual target covariance matrix and a scaled identity matrix, which helps to stabilize the alignment when the covariance matrix is ill-conditioned. The regularized target covariance matrix is computed as:


$${\text{C}}_{\text{t}\text{a}\text{r}\text{g}\text{e}\text{t},\text{r}\text{e}\text{g}}=\left(1-\lambda \right){\text{C}}_{\text{t}\text{a}\text{r}\text{g}\text{e}\text{t}}+\lambda \text{I},$$

where:

- **C**_target_: Target covariance matrix.
- **I**: Identity matrix.
- λ∈[0, 1]: Regularization parameter.

The transformation matrix for CORAL is then derived based on the regularized covariance matrix, enabling alignment of source and target data.

##### 3.4.2.2 α in EA (scaling parameter)

The α parameter in EA scales the adjustment of source and target data means to a grand mean. This parameter controls how much the data is centered around the grand mean. The alignment formula for EA is given by:


$${\text{X}}_{\text{a}\text{l}\text{i}\text{g}\text{n}\text{e}\text{d}}=\text{X}-\alpha \left(\text{M}-\text{G}\right),$$

where:

- **X**: Data matrix.
- **M**: Mean of the data.
- **G**: Grand mean of all data.
- α>0: Scaling parameter controlling the degree of mean adjustment. Small values preserve the original data distribution, while larger values (e.g., α = 100) more aggressively center data around the grand mean.

### 3.5 Canonical Correlation Analysis (CCA)

Canonical Correlation Analysis (CCA) was used as the decoding method to extract task-related features from the EEG signals. CCA identifies linear combinations of two datasets, in this case, the EEG signals (*X*) and the corresponding label-based model functions (*Y*), such that the correlation between the resulting canonical variables is maximized. The objective of CCA is to determine weight vectors *a* and *b* that solve the following optimization problem:


$$\left(a,b\right)={\text{a}\text{r}\text{g}\text{m}\text{a}\text{x}}_{a,b}\text{c}\text{o}\text{r}\text{r}\left(Xa,Yb\right),$$

where *X* represents the EEG data matrix and *Y* represents the model functions constructed using task-specific labels.

For each epoch *i*, the EEG data matrix *X*_*i*_ has dimensions 38 × 14 (time points × channels), and the model functions *Y*_*i*_ are generated based on stimulus labels. Specifically, *Y*_*i*_ is defined as:


$$\begin{array}{l} Y_{i}=y_{i}\cdot {\text{I}}_{38}, \end{array}$$ (1)

where *y*_*i*_ is the epoch-specific label (+1 for left targets and −1 for right targets), and **I**_38_ is a 38 × 38 identity matrix. The concatenated matrices *X* and *Y* are constructed across all training epochs:


$$X=\left[\begin{array}{c} X_{1}\\ X_{2}\\ \vdots \\ X_{n} \end{array}\right],\;Y=\left[\begin{array}{c} Y_{1}\\ Y_{2}\\ \vdots \\ Y_{n} \end{array}\right].$$

Once the weight vectors *a* and *b* are computed, the canonical variables *U* and *V* are obtained as:


$$\begin{array}{l} U=Xa,\;V=Yb. \end{array}$$ (2)

The canonical correlation between *U* and *V* represents the relationship between the EEG signals and the task labels. Components where the canonical correlation is statistically significant (*p* < 0.1) are retained. The spatial filters *a*, derived from CCA, are subsequently used as weights to decode EEG signals, while the canonical components *b* represent the task-related difference waves. CCA is particularly effective for analyzing EEG signals because it identifies the optimal projections that maximize the correlation between the neural activity and the task-relevant features, thereby improving the signal-to-noise ratio.

CCA was chosen because the target signal is an ERP-style, time-locked covert spatial attention response and because CCA is a well-established spatial filtering method for evoked and event-related potential decoding. Using the same general decoder family as in the original benchmark also allows the present study to isolate the effect of alignment, rather than confounding it with classifier choice.

### 3.6 Leave-one-subject-out (LOSO) cross-validation

To evaluate the generalization performance of the alignment methods and the CCA-based decoding approach, we employed the LOSO cross-validation strategy. LOSO is a robust evaluation method for transfer learning scenarios, where data from multiple subjects is available. The procedure is as follows:

1. Iteration over subjects: Each subject is iteratively designated as the target subject, while the remaining *N*−1 subjects are used as the source domain for training.
2. Source and target data: EEG data from the source subjects are concatenated end-to-end to train the decoding model, while the data of the target subject are set aside to test.
3. Alignment and parameter tuning: Alignment methods (RPA, EA, CORAL) are employed for aligning source EEG data into the domain of the target subject. Parameters such as λ for CORAL and α for EA are adjusted to get highest decoding performance on the data of the target subject.
4. Transfer learning and decoding: Target subject's data is utilized to test the CCA-based decoding model learned from the aligned source data.
5. Performance evaluation: Decoding accuracy is calculated as the percentage of correctly classified trials for the target subject. The experiment is performed for all subjects, and the final performance is averaged across iterations.

The LOSO cross-validation method is highly suitable for evaluating inter-subject transfer learning algorithms since it simulates the real scenario in which EEG data from a new subject is unavailable for training. Through gradually omitting a single subject, LOSO provides an unbiased estimate of alignment techniques' ability in subject-to-subject generalization.

Alignment parameters λ and α were optimized between the range [10^−4^, 10^4^] to maintain consistent performance. The decoding performances obtained from comparisons between alignment methods (No Alignment, RPA, EA, and CORAL) were utilized to decide the most appropriate approach to reduce inter-subject variability. Subject-specific calibration and generalization performance trade-offs are determined by this analysis as a way of appreciating the feasibility of applying feature-transformation methods in EEG-based BCIs.

**Figure 5:**
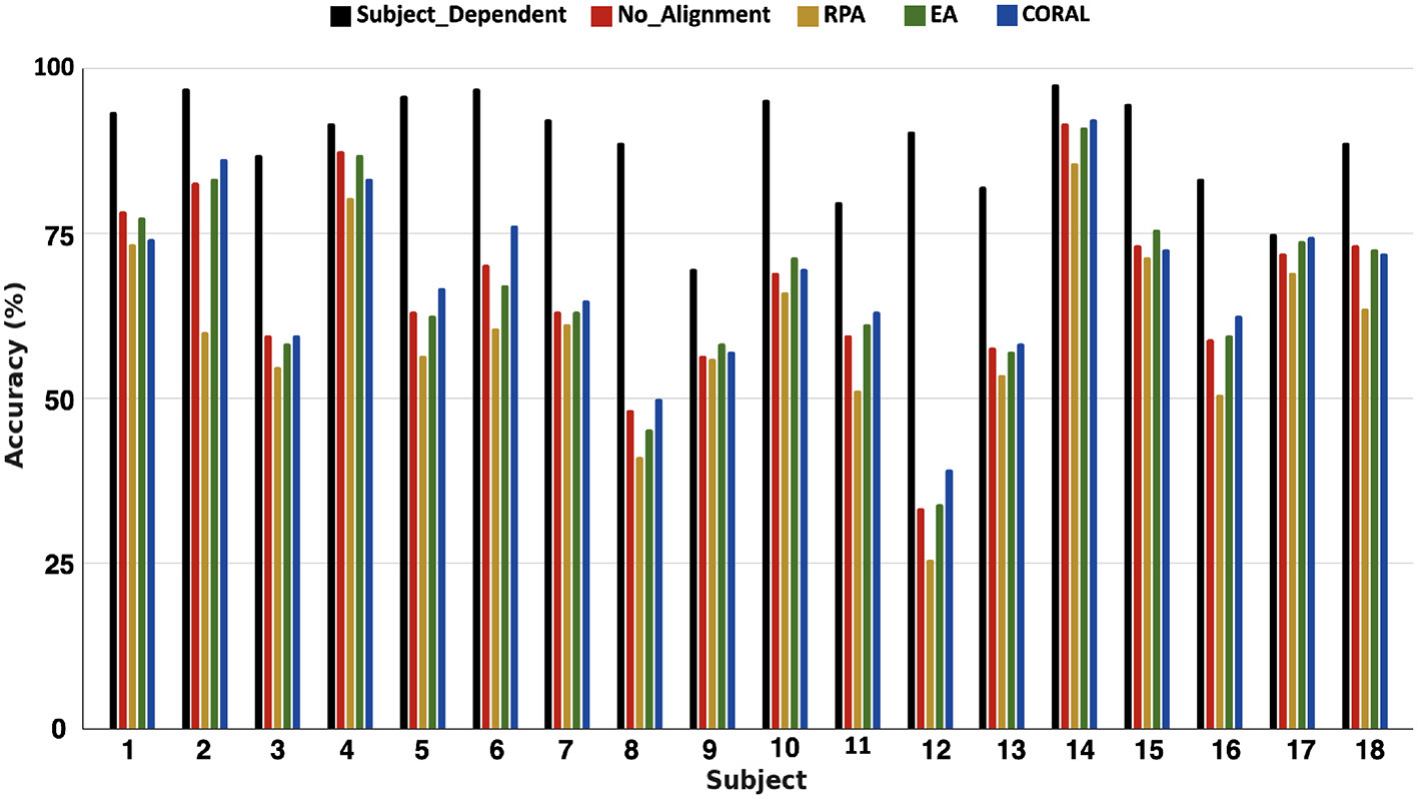
Decoding accuracy per subject for each alignment method. The figure presents accuracy for Subject-Dependent and Transfer Learning approaches including No Alignment, RPA, EA, and CORAL. The “RPA”, “EA”, and “CORAL” bars use each method's default parameterisation and are leakage-free LOSO estimates. Per-subject *tuned* accuracies (Figure 7, Tables 4, 5) are oracle upper bounds.

Parameter selection. Within each LOSO fold, alignment parameters (λ for CORAL, α for EA) are selected using the held-out target subject's labels, and the reported per-subject “best-accuracy” values therefore reflect an oracle setting. These results should be interpreted as an upper bound on subject-specific tuning rather than performance on a truly unseen subject. Aggregate fixed-parameter results (e.g., EA at α = 100), identified *post-hoc* from the same sweep, inherit the same limitation. We retain this analysis to characterize parameter sensitivity, but label it explicitly as such throughout. A leakage-free alternative would require nested LOSO with parameter selection restricted to source subjects, which we leave for future work. Default-parameter results (Figure 5) do not use target labels and are unaffected.

### 3.7 Selection and optimization of λ and α

λ and α are optimized for best decoding accuracy using LOSO cross-validation. The optimal values of parameters are selected as follows:


$$\lambda^{*}=\underset{\lambda }{\text{a}\text{r}\text{g}\text{m}\text{a}\text{x}}\text{A}\text{c}\text{c}\text{u}\text{r}\text{a}\text{c}\text{y}\left(\lambda \right),\;\alpha^{*}=\underset{\alpha }{\text{a}\text{r}\text{g}\text{m}\text{a}\text{x}}\text{A}\text{c}\text{c}\text{u}\text{r}\text{a}\text{c}\text{y}\left(\alpha \right).$$

First, the parameters were searched in the range of [10^−2^, 10^2^]. However, to ensure that the interval was sufficient, the range was incrementally expanded to [10^−4^, 10^4^]. This approach allowed for a thorough exploration of the parameter space and ensured that the optimal values for λ and α were identified without bias from the initial range. We emphasize that the optimisation described above selects parameters by maximizing accuracy on the target subject and is therefore not a leakage-safe procedure for estimating cross-subject generalization. The numbers it produces are reported in Section 4 as an oracle / sensitivity bound, not as deployable LOSO performance.

**Figure 7:**
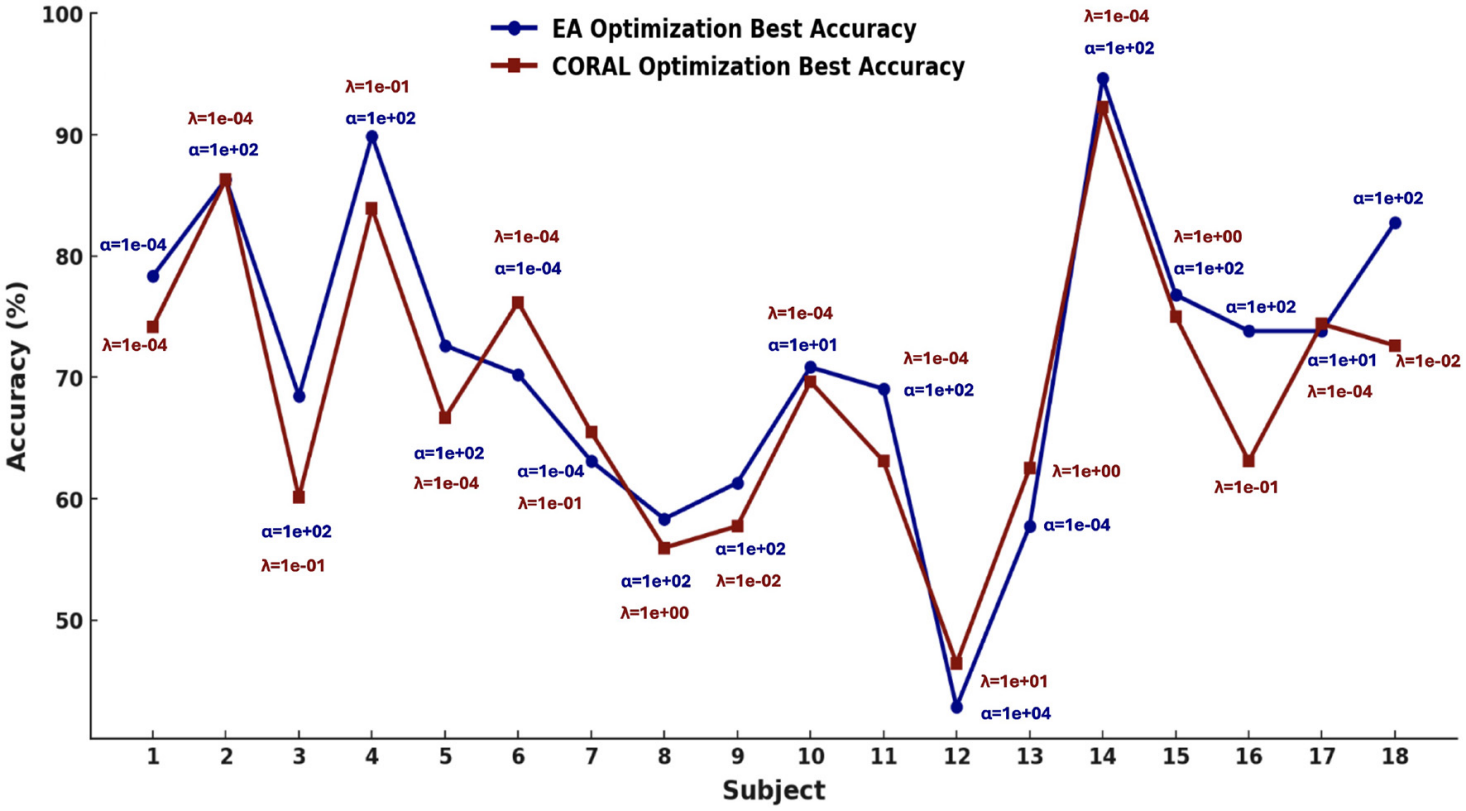
Per-subject *oracle* accuracies for EA and CORAL: for each subject and each method, the curve shows the highest accuracy attained across the full parameter sweep, with the corresponding λ or α annotated. Because the parameter is chosen by inspecting the target subject's own decoding accuracy, these values are an upper bound on what subject-specific tuning could achieve and are *not* a leakage-safe estimate of generalization to a new subject. Comparison of best accuracy achieved by EA Optimization (blue line with circular markers) and CORAL Optimization (red line with square markers) across 18 subjects. The x-axis shows subject IDs and the y-axis shows accuracy percentages. Optimal parameter values for each subject are annotated.

**Table 3:** Asymptotic computational cost of each pipeline component.

Stage	Dominant operation	Asymptotic cost
Pre-processing	Filtering, resampling, epoching	*O*(*N*·*C*·*T*_raw_) (one-time per subject)
Euclidean Alignment (EA)	Mean subtraction	*O*((*N*_src_+*N*_tgt_)·*C*·*W*·*S*)
CORAL	Covariance + eigendecomposition	$O\left(\left(N_{\text{s}\text{r}\text{c}}+N_{\text{t}\text{g}\text{t}}\right)\cdot C^{2}\cdot W\cdot S\right)+O\left(C^{3}\right)$
RPA	Covariance + eigendecomposition	$O\left(\left(N_{\text{s}\text{r}\text{c}}+N_{\text{t}\text{g}\text{t}}\right)\cdot C^{2}\cdot W\cdot S\right)+O\left(C^{3}\right)$
CCA training	singular value decomposition (SVD) on stacked source data	$\sim O\left(\left(N_{\text{s}\text{r}\text{c}}WS\right)\cdot \min{\left(W,C\right)}^{2}\right)$
Online inference	Projection + correlation	*O*(*W*·*S*·*C*·*k*)

**Table 4:** CORAL optimization accuracy (%).

Subject	λ **Lambda values**								
	0.0001	0.001	0.01	0.1	1	10	100	1,000	10,000
1	**74.17**	**74.17**	73.33	71.67	70.83	60.83	61.67	62.50	62.50
2	**86.31**	**86.31**	**86.31**	**86.31**	77.38	69.64	71.43	72.62	72.02
3	59.52	59.52	58.93	**60.12**	56.55	48.81	49.40	48.81	48.81
4	83.33	83.33	83.33	**83.93**	**83.93**	44.05	49.40	51.79	51.79
5	**66.67**	**66.67**	**66.67**	66.07	52.98	60.12	60.12	60.12	60.12
6	**76.19**	**76.19**	**76.19**	75.00	61.31	61.90	62.50	63.10	63.10
7	64.88	64.88	64.88	**65.48**	64.29	48.81	52.98	52.38	52.38
8	50.00	50.00	50.00	50.00	**55.95**	52.38	53.57	53.57	52.98
9	57.14	57.14	**57.74**	57.14	52.98	43.45	45.83	45.83	45.83
10	**69.64**	**69.64**	**69.64**	69.05	60.12	64.88	64.29	65.48	64.88
11	**63.10**	**63.10**	**63.10**	61.90	**63.10**	57.74	58.93	57.74	56.55
12	39.29	39.29	39.29	39.29	36.31	**46.43**	45.83	44.64	45.24
13	58.33	58.33	58.33	58.33	**62.50**	53.57	55.36	54.76	54.17
14	**92.26**	**92.26**	**92.26**	**92.26**	88.69	64.29	67.26	66.07	66.67
15	72.62	72.62	72.62	72.02	**75.00**	57.14	55.95	54.76	54.76
16	62.50	62.50	62.50	**63.10**	55.95	49.40	51.19	51.19	52.38
17	**74.40**	**74.40**	**74.40**	**74.40**	69.05	45.83	54.76	48.21	44.64
18	72.02	72.02	**72.62**	72.02	67.86	50.00	46.43	46.43	46.43

Bold blue values indicate the best accuracy per subject. These are oracle/sensitivity values; see Section 3.6.

**Table 5:** EA optimization accuracy (%).

Subject	α **Alpha values**								
	0.0001	0.001	0.01	0.1	1	10	100	1,000	10,000
1	**78.33**	**78.33**	**78.33**	**78.33**	**78.33**	77.50	**78.33**	47.50	37.50
2	82.74	82.74	82.74	82.74	82.74	82.74	**86.31**	52.38	50.60
3	59.52	59.52	59.52	59.52	59.52	58.33	**68.45**	48.81	44.64
4	87.50	87.50	87.50	87.50	87.50	85.12	**89.88**	51.79	47.02
5	63.10	63.10	63.10	63.10	63.10	60.71	**72.62**	50.00	47.02
6	**70.24**	**70.24**	**70.24**	**70.24**	**70.24**	**70.24**	66.67	47.62	44.64
7	**63.10**	**63.10**	**63.10**	**63.10**	**63.10**	58.33	59.52	42.26	42.86
8	48.21	48.21	48.21	48.21	48.21	51.19	**58.33**	51.19	51.19
9	56.55	56.55	56.55	56.55	57.14	57.74	**61.31**	54.76	55.95
10	69.05	69.05	69.05	69.05	70.24	**70.83**	66.67	60.12	54.76
11	59.52	59.52	59.52	59.52	60.71	62.50	**69.05**	55.95	54.17
12	33.33	33.33	33.33	33.33	32.14	31.55	28.57	40.48	**42.86**
13	**57.74**	**57.74**	**57.74**	57.14	56.55	56.55	54.76	54.76	52.98
14	91.67	91.67	91.67	91.67	91.67	91.67	**94.64**	54.76	51.19
15	73.21	73.21	73.21	73.21	73.21	73.81	**76.79**	56.55	55.95
16	58.93	58.93	58.93	58.93	58.93	58.33	**73.81**	60.12	55.95
17	72.02	72.02	72.02	72.02	72.02	**73.81**	71.43	53.57	50.00
18	73.21	73.21	73.21	73.21	73.21	73.21	**82.74**	54.76	51.79

Bold blue values indicate the best accuracy per subject. These are oracle/sensitivity values; see Section 3.6.

### 3.8 Impact of λ and α

- A low λ (λ → 0) in CORAL emphasizes the original covariance structure of the target domain, while a high λ (λ → 1) leads to an isotropic covariance matrix, simplifying the data structure.
- A low α (α → 0) in EA maintains the original data distribution, while a high α (α → 1) completely adjusts the data to the grand mean, enhancing alignment but potentially losing individual differences.

### 3.9 Computational cost

We characterize the asymptotic cost of each pipeline component, as BCI deployment imposes runtime constraints. Let *C* = 14 denote the number of channels, *W* = 38 samples per epoch (after filtering and 50 Hz resampling), *S* = 10 epochs per trial, *N*_src_ the number of source trials (≈2856 per LOSO fold), and *N*_tgt_ = 168 target trials. The dominant operations are summarized in Table 3.

With *C* = 14, cubic terms (*O*(*C*^3^)) are negligible, and all alignment methods scale effectively linearly with the number of trials. The dominant cost within a LOSO fold is CCA training on stacked source data. At inference, only a fixed-size projection and correlation are required, independent of source dataset size, as alignment and CCA parameters are pre-computed offline. Thus, the pipeline incurs a one-time calibration cost that scales with available data and a lightweight per-trial cost compatible with real-time BCI deployment.

We do not report wall-clock timings, as the goal here is to establish asymptotic behavior; hardware-specific benchmarks are left for future work.

## 4 Results

### 4.1 Decoding accuracy per subject for each alignment method

Figure 5 illustrates the decoding accuracy for each subject using five distinct approaches: Subject-Dependent *vs*. Transfer Learning (baseline: No Alignment *vs*. using the alignment method: RPA, EA, and CORAL). The bar plot highlights the variability in performance across subjects and demonstrates the comparative effectiveness of each alignment method. In addition, for each subject, the best method and second-best method are identified based on the highest and second-highest accuracy, respectively. The table illustrates the performance differences between subject-dependent decoding and transfer learning methods, highlighting how well transfer learning approaches generalize to individual subjects. The results suggest that CORAL frequently emerges as the best-performing alignment method for many subjects.

Here are some key observations and deductions from Figure 5:

#### 4.1.1 Subject-dependent *vs*. transfer learning methods

Subject-dependent decoding outperforms all transfer learning methods for most subjects. This is expected since subject-dependent decoding has access to the subject's own data for both training and testing, unlike transfer learning methods that train on other subjects.

#### 4.1.2 Best and second-best methods

The CORAL method is frequently the best alignment method, appearing as the top method for 9 out of 18 subjects. The second-best method varies across subjects but EA appears often as the second-best method.

#### 4.1.3 Subject-specific performance

- For some subjects (like 4, 14, and 17), the transfer learning methods perform relatively close to the subject-dependent method (less than 10% difference), suggesting that for certain subjects, transfer learning methods can generalize well.
- Certain subjects (like Subjects 3, 5, 7, 8, and 12) have very high Subject-Dependent accuracy, which is significantly higher (by more than 25% difference) than the best transfer learning method.
- For the remaining subjects, the difference between Subject-Independent and Transfer learning is between 10% and 25%.

#### 4.1.4 Impact of variability across subjects

The differences in accuracy for each subject highlight the role of individual differences in EEG data. The performance of alignment methods varies significantly, indicating that some subjects may have signals that are more “compatible” with the transfer learning models.

#### 4.1.5 Generalization

CORAL emerges as a consistently strong performer for transfer learning across subjects. The variability in the best and second-best methods across subjects suggests that no single alignment-based transfer learning method is optimal for all subjects.

**Figure 6:**
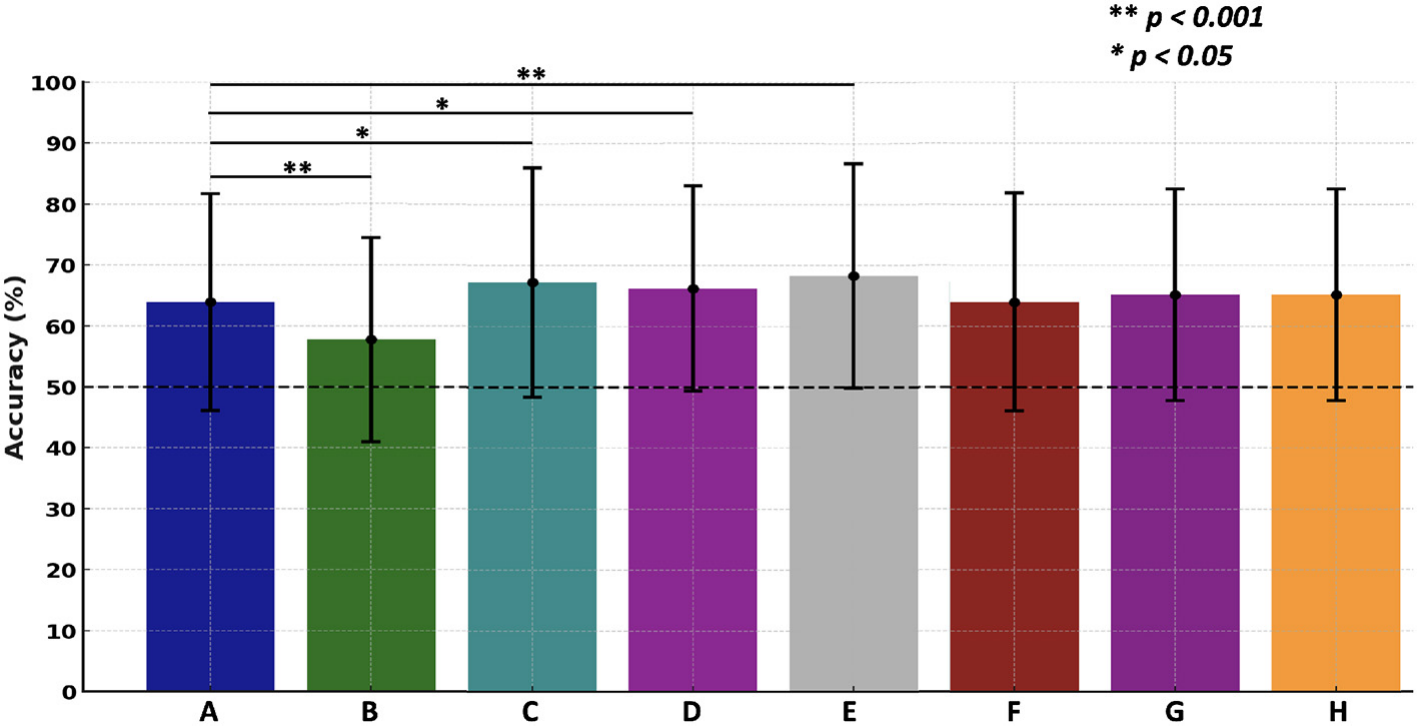
Mean leave-one-subject-out classification accuracy across alignment settings. Bars show the mean across 18 subjects and error bars show standard deviation. The dashed line marks chance level at 50%. Asterisks indicate paired comparisons against No Alignment. Methods: **(A)** No Alignment, **(B)** RPA, **(C)** EA optimization (α = 100), **(D)** Optimized CORAL (per-subject best λ), **(E)** Optimized EA (per-subject best α), **(F)** EA, **(G)** CORAL, **(H)** CORAL optimization (λ = 0.001).

The classification accuracy of various alignment methods was compared against the “No Alignment” baseline, as shown in Figure 6. The mean accuracy for each method is represented by the height of the bars, with standard deviations visualized as error bars. The dashed horizontal line at 50% indicates the chance level, labeled for clarity. Significance levels, determined via paired t-tests between each method and the baseline, are marked above the bars (** for *p* < 0.001 and * for *p* < 0.05). The alignment methods evaluated include RPA, EA, CORAL, and their optimized variants (e.g., CORAL Optimization (λ = 0.001) and EA Optimization (α = 100)), alongside versions tuned for best accuracy. Results demonstrate variability across methods, with several achieving significant improvements over the baseline. This highlights the potential of alignment techniques in enhancing classification performance.

For EA (α = 100) *vs*. No Alignment, the standardized paired effect size is Cohen's *d*_*z*_≈0.59 (mean improvement 3.44%, *s*_*d*_ = 5.88), indicating a medium effect. The corresponding test yields *t*(17)≈2.48 (*p*≈0.024), with a 95% confidence interval of [0.52%, 6.36%]. Although strictly positive, the interval suggests a modest population-level gain. In practical terms, a 3.44% gain corresponds to ~6 additional correctly decoded trials per subject (168 trials total), implying a small but consistent increase in information transfer rate. We also report the number of subjects showing improvement, no change, or degradation under EA (α = 100) relative to No Alignment.

### 4.2 Performance optimization for RPA alignment method

The experimental results indicate that the best performance for rRPA is achieved when α is close to 1. This suggests that RPA may not be providing effective alignment, as the covariance structure is almost entirely replaced by an identity matrix scaled to the average variance. The implications are as follows:

- Loss of covariance information: Using an identity matrix eliminates all covariance structure in the data.
- Alignment ineffectiveness: The whitening and alignment steps in RPA are rendered ineffective since the covariance matrices for source and target data become proportional to identity matrices.
- No meaningful transformation: The alignment matrix **T**_RPA_ becomes a scalar multiple of the identity matrix, failing to transform the source data meaningfully.

These findings highlight the limitations of RPA in aligning the data effectively and motivate further exploration of optimization strategies for CORAL and EA alignment methods.

### 4.3 Performance optimization for CORAL and EA alignment methods

Figure 7 compares the decoding accuracy achieved by the CORAL and EA alignment methods across 18 subjects. The regularization parameter λ for CORAL and scaling parameter α for EA corresponding to the best accuracy are annotated for each subject.

- Performance trends: Both CORAL and EA methods exhibit variability in decoding accuracy across the 18 subjects. CORAL slightly outperforms EA for certain subjects (e.g., 3 and 13), while EA achieves higher accuracy for others (e.g., 2 and 16).
- Parameter values:
  - For CORAL, the optimal regularization parameter λ is generally small (λ = 0.0001 or 0.01), indicating minimal regularization provides better alignment in most cases.
  - For EA, the optimal scaling parameter α tends to be high (α = 100), suggesting that aligning data closely to the grand mean enhances decoding performance.
- Subject-specific patterns: The figure reveals that no single λ or α value works universally across all subjects, emphasizing the importance of subject-specific optimization for alignment parameters.
- Insights on method performance:
  - CORAL leverages covariance regularization effectively to address mismatched data distributions.
  - EA relies on higher scaling to improve alignment by centralizing data around the grand mean.
- Practical implications: The analysis highlights the necessity of optimizing λ and α using cross-validation. Expanding the search intervals for these parameters ([10^−4^, 10^4^]) ensured robust coverage of potential values, resulting in meaningful improvements in alignment performance.

### 4.4 Optimization results

Tables 4, 5 present a parameter-sensitivity sweep. Each entry corresponds to the LOSO accuracy obtained when selecting that parameter with access to the target subject's labels. Consequently, the per-subject “best” values are oracle quantities, and comparisons across parameter settings reflect sensitivity to hyperparameters rather than leakage-free generalization performance.

Tables 4, 5 summarize the decoding accuracies achieved using the CORAL and EA optimization approaches, respectively, across different parameter values for each subject.

#### 4.4.1 CORAL optimization results

For the CORAL method, the parameter λ was varied across a wide range of values (λ∈{0.0001, 0.001, 0.01, 0.1, 1, 10, 100, 1000, 10000}). The results indicate that lower values of λ generally lead to higher decoding accuracies, with the peak performance for most subjects occurring within the range λ ≤ 0.1.

- Effect of lambda on accuracy: For many subjects, the decoding accuracy stabilizes as λ increases, with the optimal performance often observed at lower λ values.
- Consistency across subjects: Some subjects show stable accuracy across the λ range, while others exhibit sensitivity.
- Peaks indicate best performance: The optimal λ values for each subject are indicated by the peaks in their respective accuracy trends.

#### 4.4.2 EA optimization results

For the EA method, the parameter α was varied across a similar range α∈[10^−4^, 10^4^] tend to yield the highest decoding accuracies for most subjects. Notably, as α increased beyond 1000, a sharp decline in accuracy was observed for all subjects.

- Effect of alpha on accuracy: EA optimization demonstrates strong sensitivity to α, particularly for small values (e.g., α < 1).
- Deterioration with increased alpha: As α increases, the accuracy will plateau or decline marginally.
- Variability of subjects: Subject-specific parameter tuning is required.

#### 4.4.3 General observations

- Both methods exhibit strong sensitivity to their corresponding parameters at smaller values.
- EA experiences more precipitous accuracy changes with variation in α, compared to the smoother patterns of CORAL.
- Subject-specific optimization is vital to achieve maximum decoding accuracy.

These observations highlight the need for optimizing alignment parameters for every individual to get the best possible performance for EEG-based decoding applications.

### 4.5 Why the optimal method differs across subjects

The three alignment operators address distinct forms of source–target mismatch. EA primarily corrects *first-order* (mean-level) differences by shifting signals toward a common grand mean, whereas CORAL and RPA target *second-order* (covariance) structure, with RPA operating at the per-trial level and thus more sensitive to variability. This distinction helps explain several patterns in the results. First, subjects with low subject-dependent accuracy (e.g., Subject 12 in Figure 5) also perform poorly under all transfer methods, indicating that target signal quality, rather than alignment, is the limiting factor. Second, the optimal EA scaling (Tables 4, 5) is bimodal: most subjects favor either α≈10^−4^ (minimal adjustment) or α = 100 (strong centring), suggesting two regimes depending on how far the subject mean deviates from the cohort mean. Third, CORAL performs best with small λ (10^−4^ to 10^−1^) and degrades for larger values, indicating that the empirical target covariance contains informative structure that is disrupted by excessive regularization.

Overall, no single alignment method is universally optimal because subjects differ in which component of mismatch—mean or covariance—dominates.

### 4.6 Limitations

This study has several limitations. First, evaluation is restricted to a single covert spatial attention dataset (18 subjects), limiting generalizability to other paradigms such as motor imagery or alternative ERP tasks. Second, we focus on classical alignment methods and do not benchmark recent deep transfer, self-supervised, or foundation-model approaches, which operate under different data and computational regimes.

Third, the per-subject parameter sweeps (Section 4) and the fixed α = 100 result (Figure 6) are not leakage-safe: parameters are selected based on the held-out target subject's accuracy and thus constitute an oracle upper bound on subject-specific tuning. A leakage-free evaluation would require nested LOSO with parameter selection restricted to source subjects, which we identify as the primary direction for future work. In contrast, the default-parameter results (No Alignment, RPA, EA, CORAL; Figure 5) do not use target labels and remain unbiased.

Finally, the 1.0–12.5 Hz pass-band, adopted from Reichert et al. (2020), is well-matched to the N2pc and posterior alpha signatures of covert spatial attention but excludes higher-frequency activity that may be informative in other paradigms. Evaluating broader spectral ranges is left for future work.

## 5 Discussion and conclusion

In order to achieve the full potential of alignment methods described in this study, such as CORAL and EA, it is highly recommended to get calibration data from the subject of interest. The necessity and specificity of such calibration data depend on several critical factors that affect performance and implementation. As for target subject calibration data, it is important to allow alignment methods to learn the particular statistical properties of the EEG signals of the target subject, such as individual variability, noise, and brain activity patterns, which means improved alignment performance. A notable finding is that even a small amount of calibration data, such as a few minutes of EEG recordings, can enhance the alignment accuracy. Task-specific calibration becomes a factor to consider. Ideally, the calibration data should be task-specific, as EEG signals highly depend on the task being performed. For instance, motor imagery calibration data is not likely to generalize well to emotion recognition tasks. In cases where task-specific data is unavailable, resting-state EEG or unrelated tasks may still provide a useful baseline for alignment, albeit with reduced performance compared to task-specific calibration. Implementation scenarios present different approaches based on data availability. With calibration data, a small amount of task-specific recordings can be collected from the target subject before alignment, used to fine-tune the alignment parameters. In scenarios where calibration data cannot be collected, alignment can rely on average transformations derived from source subjects. While this approach is less effective, it allows for real-world applications with minimal setup requirements. Real-world implications vary across applications. In clinical settings, calibration data is often feasible as patients can provide task-specific EEG recordings during initial sessions. For consumer-grade applications, such as wearable devices or gaming applications, task-specific calibration may not be practical. Adaptive or semi-automated recalibration methods could be a viable solution in these scenarios. Real-time systems can incorporate dynamic recalibration, collecting data incrementally during use to continuously improve alignment performance without requiring extensive pre-session calibration. These findings suggest that obtaining calibration data, preferably task-specific, is essential for maximizing the performance of alignment methods. However, practical constraints in real-world applications necessitate further research into automated and adaptive calibration techniques.

In this work, we demonstrated that alignment methods can improve EEG-based BCI performance through optimized parameter selection. The EA Optimization (α = 100) method achieved an average improvement of 3.4% (*SD* = 5.88) compared to the baseline (No Alignment), showing a statistically significant difference (*p* < 0.05, paired t-test). These results indicate the promise of optimized alignment methods for improving cross-subject transfer learning in EEG-based BCI systems. The study indicates that optimal results are generated by task-specific calibration data, yet in reality, practical implementations should weigh performance demands against real-world requirements. The findings indicate that CORAL is best with low values (λ ≤ 0.1), whereas EA achieves its best fixed-parameter performance at α = 100, with subject-specific optimization sometimes favoring other values.

The reported 3.4% improvement is based on a parameter (α = 100) selected from the same per-subject sweep used to compute the accuracies, and thus represents an upper bound under favorable tuning rather than deployable performance. A leakage-free estimate would require nested LOSO evaluation, which we identify as immediate future work.

The practical implications of these findings extend beyond the lab, suggesting directions for more flexible and accessible BCI systems. With continued advances in the technology, the merging of sophisticated calibration techniques with robust alignment methods will be critical to bridging this gap between theoretical capabilities and practical use, particularly in scenarios requiring accelerated deployment and flexibility across different subjects and tasks.

## Data Availability

The original contributions presented in the study are included in the article/supplementary material, further inquiries can be directed to the corresponding authors.
